# Prospective Randomized Trial Comparing Hepatic Venous Outflow and Renal Function after Conventional versus Piggyback Liver Transplantation

**DOI:** 10.1371/journal.pone.0129923

**Published:** 2015-06-26

**Authors:** Marília D’Elboux Guimarães Brescia, Paulo Celso Bosco Massarollo, Ernesto Sasaki Imakuma, Sérgio Mies

**Affiliations:** Laboratório de Anatomia Médico-Cirúrgica (LIM-02), Departamento de Cirurgia, Faculdade de Medicina da Universidade de São Paulo, São Paulo, Brazil; Clínica Universidad de Navarra, SPAIN

## Abstract

**Background:**

This randomized prospective clinical trial compared the hepatic venous outflow drainage and renal function after conventional with venovenous bypass (n = 15) or piggyback (n = 17) liver transplantation.

**Methods:**

Free hepatic vein pressure (FHVP) and central venous pressure (CVP) measurements were performed after graft reperfusion. Postoperative serum creatinine (Cr) was measured daily on the first week and on the 14^th^, 21^st^ and 28^th^ postoperative days (PO). The prevalence of acute renal failure (ARF) up to the 28th PO was analyzed by RIFLE-AKIN criteria. A Generalized Estimating Equation (GEE) approach was used for comparison of longitudinal measurements of renal function.

**Results:**

FHVP-CVP gradient > 3 mm Hg was observed in 26.7% (4/15) of the patients in the conventional group and in 17.6% (3/17) in the piggyback group (p = 0.68). Median FHVP-CVP gradient was 2 mm Hg (0–8 mmHg) vs. 3 mm Hg (0–7 mm Hg) in conventional and piggyback groups, respectively (p = 0.73). There is no statistically significant difference between the conventional (1/15) and the piggyback (2/17) groups regarding massive ascites development (p = 1.00). GEE estimated marginal mean for Cr was significantly higher in conventional than in piggyback group (2.14 ± 0.26 vs. 1.47 ± 0.15 mg/dL; p = 0.02). The conventional method presented a higher prevalence of severe ARF during the first 28 PO days (OR = 3.207; 95% CI, 1.010 to 10.179; p = 0.048).

**Conclusion:**

Patients submitted to liver transplantation using conventional or piggyback methods present similar results regarding venous outflow drainage of the graft. Conventional with venovenous bypass technique significantly increases the harm of postoperative renal dysfunction.

**Trial Registration:**

ClinicalTrials.gov https://clinicaltrials.gov/ct2/show/NCT01707810

## Introduction

A technical aspect of great importance in liver transplantation (LTx) is the maintenance of the hemodynamic status of the recipient during the anhepatic phase. In the conventional technique, the liver is removed *en bloc* with the retrohepatic portion of the inferior vena cava (IVC), interrupting the venous return of the infradiaphragmatic bed. In order to overcome the consequences of this maneuver, a temporary venovenous bypass (VVB) driven by a centrifugal pump is usually used, allowing diversion of the IVC blood flow to the superior vena cava [1]. Despite hemodynamic advantages, VVB presents risks such as air and thrombotic pulmonary embolism and formation of lymphatic fistulas in the axillary and inguinal regions, where incisions for the placement of catheters are performed [2–4]. In addition, there is the inconvenience of pump, circuit and equipment operation costs.

Since late nineties, the piggyback method of LTx became increasingly used [2,5]. This technique consists of removing the diseased liver with preservation of the retrohepatic portion of the IVC [6,7]. Thus, flow through IVC can be maintained, eliminating the need for VVB [8]. In this method, reconstitution of the venous drainage path is performed by means of an anastomosis between the suprahepatic portion of donor’s IVC and the ostium of the recipient’s hepatic veins. An alternative is a side-to-side (SS) anastomosis between the retrohepatic portion of graft’s and recipient’s IVC [9,10].

On comparing with the conventional method, piggyback transplantation allowed greater hemodynamic stability, lower consumption of blood components, shorter warm ischemia time and lower hospital charges [4,11–13]. In contrast to these advantages, there are some inconveniences such as a specific difficulty in venous drainage of the liver [14–23]. Although there are several reports on this complication, the incidence of venous outflow block in patients operated by using the piggyback method has discrepant results, varying from zero to 40% [17,20]. However, some of these studies do not present a control group and none specifically approaches this issue by means of a clinical randomized prospective trial.

Studies on alterations in hepatic venous outflow after LTx generally use two distinct evaluation criteria. In some of them, venous outflow block is hemodynamically defined by the presence of a significant pressure gradient between hepatic veins and right atrium, even without apparent manifestations [17]. In others, the diagnosis is mainly based on the development of clinical signs suggesting this complication, such as the intraoperative aspect of the graft [16,24], development of post-LTx ascites [25,26], and acute renal failure (ARF) [27,28]. It is important to note that the frequency of venous outflow block may be quite different depending on the used approach. Additionally, it should be emphasized that the development of ARF after LTx may be related to several pre-, intra- and postoperative conditions, such as Model for End-Stage Liver Disease (MELD) score, basal renal function, hemodynamic status, red blood cells requirements, surgical technique, use of nephrotoxic drugs, graft dysfunction and sepsis [29–33]. The concurrent influence of these factors turns difficult the identification of the role played by the outflow block in this complication.

Moreover, postoperative ARF is suspected to be associated to conventional or piggyback techniques apart from detectable hepatic venous drainage disturbances [34]. However, in a previous retrospective study performed by our group, no relationship was identified between surgical technique and ARF [30]. Indeed, although Cabenzuelo et al. [34], in 2003, have identified conventional technique as a predictive factor for development of ARF, the same group reported a further investigation on their data [31], in 2006, when piggyback method was found to significantly reduce postoperative ARF in univariate analysis but did not appear as an independent factor in multivariable logistic regression. Due these conflicting results, it’s still considered an open question whether piggyback method allow for a reduction of renal dysfunction after LTx [32].

The present study is a prospective randomized clinical trial designed to compare the hepatic venous outflow between patients transplanted by the conventional or the piggyback method. Additionally, postoperative renal function was compared between the two methods of LTx.

## Patients and Methods

The original protocol was submitted to the Ethics Committee of the Institution (CAPPesq—Comissão de Ética para Análise de Projetos de Pesquisa do Hospital das Clínicas da Faculdade de Medicina da Universidade de São Paulo) with two parallel arms that were conducted and reported together: the first (#675/98), regarding the analysis of hepatic venous outflow drainage after conventional with VVB or piggyback LTx as the primary outcome measure, was approved on December 9, 1998 [S1 and S2 Protocols]; the second arm (#743/98), regarding the study of postoperative renal function in the same population, was approved on January 13, 1999 [S3 and S4 Protocols]. All clinical investigation was conducted according to the principles expressed in the Declaration of Helsinki. Informed written consent was obtained from each patient.

Thirty-two patients recruited among LTx recipients submitted to surgery at the Hospital das Clínicas da Faculdade de Medicina da Universidade de São Paulo between October 8, 1999 and September 30, 2000 were studied and followed up until June 30, 2006. Only after completion date, this trial was registered at the ClinicalTrials.gov site of the U.S. National Institutes of Health, on October 12, 2012 (Identifier # NCT01707810). The reason for the delay in registering this study was that the research was commenced before the International Committee of Medical Journal Editors, in 2005, and World Health Organization, in 2006, implemented a policy recommending prospective registration of all clinical trial. The authors state that there are no unregistered ongoing related trials for this intervention in our group.

The patients were assigned by randomization to two groups: LTx performed using the conventional method with VVB or the piggyback method. Most, but not all of these patients, was shared with 3 previously reported clinical trials that compared distinct outcomes variables between the conventional with VVB and the piggyback methods of LTx: pulmonary alterations [35], bacterial translocation [36], and inflammatory cytokines production [37].

Inclusion criteria admitted: both genders, age 18 years or older, first elective LTx with no clinical or technical reasons justifying a preferential option by conventional or piggyback method. For this reason, we excluded patients submitted to living donor LTx, in whom IVC is routinely preserved, and those with familial amyloidotic polyneuropathy, whom, at the time of recruitment, were routinely submitted to conventional LTx in our service [38,39]. Eligibility assessment and enrollment were conducted by two transplant nursing coordinators along with one of the authors (P.C.B.M.). Consent to participate in the study was obtained from each patient before admission for the transplant surgery. Child-Pugh and Mayo’s MELD score [40,41], without considering liver disease etiology, were calculated using results of blood samples collected immediately before surgery. The randomization process and the assignments of participants were performed after the induction of the anesthesia by a group of physiotherapists and nurses not involved with the intraoperative care of the patients. In each case, randomization was paired according to the Child-Pugh’s Score. Stratification was performed by blocking randomization in each subset of patient (scores A, B, or C), with blocks of size 2 with a one-to-one allocation ratio. Randomization was obtained by “coin-tossing” in order to facilitate pairing process at the surgical theater. Recruitment was interrupted after at least 15 patients were effectively studied in each group.

Perioperative care was delivered by using the same standard protocol in all cases. All anesthetic procedures were conducted by a dedicated liver transplant anesthesia team, composed by three anesthesiologists. Three surgeons performed all recipient operations and early postoperative care was conducted on a specific liver transplant intensive care unit. All patients were entered into a triple immunosuppression protocol with cyclosporine, corticosteroids and azathioprine.

The following donor variables were registered: age, gender, weight, diagnosis, and geographic sharing (local, if the graft is procured in the metropolitan area of the city of São Paulo; regional, if collected in the state of São Paulo; or national, if obtained from any other location in Brazil).

Recipient operations were performed with small adaptations in the standard technique in order to evaluate the pressure gradient between the hepatic vein and the right atrium in the two groups. Free hepatic vein pressure (FHVP) was measured using an 8F polyethylene catheter with a multiperforated distal end, which was introduced into the graft’s right hepatic vein for at least 6 cm during *ex situ* preparation on the back table. The proximal end of this catheter was exteriorized in the infrahepatic portion of the graft IVC.

In the conventional method, the infra- and suprahepatic portions of the IVC were cross-clamped during the anhepatic phase and the IVC and portal venous return was maintained by a portal-femoral-axillary VVB driven by a centrifugal pump (BP = 80 Bio-Pump and Bio-Console 450 Pump Speed Controller; Medtronic Bio-Medicus, Minneapolis, MN, USA). In these cases, IVC reconstruction was performed by end-to-end anastomosis above and below the liver. In the piggyback method, IVC was not cross-clamped in any case. Implantation method of the graft IVC on the recipient IVC was not standardized, being defined during the procedure by the surgeon in charge. In the two groups, all patients were submitted to simultaneous arterial and portal revascularization, according to the routine of the service [42].

In the conventional group, the proximal end of the right hepatic vein catheter was exteriorized through the infrahepatic IVC anastomosis. In the piggyback group, infrahepatic IVC was ligated around the catheter. Central venous pressure (CVP) was obtained using a Swan-Ganz catheter (routine procedure). Measurement of hepatic vein and right atrium pressure was made once, after concluding biliary anastomosis.

All measurements were obtained with the same transducer, using the mid-axillary line as zero reference level. Pressure measurements were performed in apnea, in order to avoid oscillations in the pressure curve determined by patient’s respiratory incursions. When oscillations persisted in spite of this maneuver, the arithmetic mean of the observed maximum and minimum pressure values was recorded. “Hepatic venous outflow block” was considered when a FHVP-CVP gradient higher than 3 mm Hg was verified [17,19,43].

Postoperative massive ascites was arbitrarily defined as abdominal fluid accumulation with a volume over than 500 mL/day for more than 30 days evaluated through body weight, abdominal drain output or paracentesis.

Serum creatinine (Cr) was determined in the preoperative period (immediately before surgery), on postoperative days (PO) 1 to 7 and on the 14^th^, 21^st^ and 28^th^ PO. The prevalence of ARF was analyzed according to the RIFLE-AKIN criteria [44,45]. Based on changes in Cr up to the 28^th^ PO from the baseline condition, patients were daily classified in four grades of increasing ARF severity: no renal impairment (Class N); “Risk” (Class R), if Cr increased from 50% to 100% (1.5- to 2-fold); “Injury” (Class I), if increasing was more than 100% to 200% (> 2- to 3-fold); and “Failure” (Class F), if Cr increase was higher than 200% (> 3-fold) or Cr was higher than or equal to 4.0 mg/dL with an acute increase of at least 0.5 mg/dL. Patients requiring renal replacement therapy were classified as Class F irrespective of Cr values.

### Statistical Analysis

The minimum sample size was estimated at 30 patients, intending to detect a difference of at least 1.0 mm Hg between the FHVP-CVP gradient of the conventional and piggyback groups, with an estimated standard deviation of 0.9 mm Hg [17]. With the proposed sample of 30 subjects divided in two groups and setting the significance level at 5% (α = 0.05), the study would have an estimated power of 84% to yield a statistically significant result. Regarding postoperative overall Cr, this sample would be sufficient to detect a difference of at least 1.1 mg/dL between the two surgical methods, with an estimated power of 83%, considering an expected standard deviation of 1.0 mg/dL [30].

Normally distributed quantitative variables were compared by Student’s *t* test, ANOVA or Bonferroni’s multiple comparison method. When normality could not be assumed, Mann-Whitney test was used. Qualitative variables were compared by Yates-corrected Pearson’s chi-squared test, when no expected frequency was less than 5. When this situation occurred, Fisher’s exact test was used. Patient’s survival was calculated constructing actuarial Kaplan-Meier curves and compared using the Cox-Mantel (log-rank) test [46,47].

A Generalized Estimating Equation (GEE) approach was used for comparison of longitudinal measurements of renal function [48,49]. For statistical analysis of ARF severity based on RIFLE-AKIN criteria, this response variable was dichotomized by unifying Class N with Class R (no/mild ARF), and Class I with Class F patients (severe ARF). Variables included in the model were the PO day (within-subject variable), the surgical method (conventional or piggyback), and either Cr or severe ARF prevalence (numeric or binary dependent response variables). The possibility of correlation among repeated measures over time was considered by using five different candidate working correlation structures: unstructured, independent, exchangeable, m-dependent and first-order autoregressive. The smallest Quasi-Likelihood under Independence Model Criterion (QIC) value was used for selecting the best correlation structure [50].

Statistical analysis was performed using the software Sample Power version 9.0 and IBM SPSS Statistics version 21.0. A 5% significance level was used. Values are expressed as means ± standard deviation, estimated marginal means ± standard error, Odds Ratio (OR) and 95% confidence interval (95% CI).

### Deviations from the Original Trial Protocol

Some deviations from the original trial protocol occurred during the conduction of the research. The estimated minimum sample size was reduced from 42 to 30 subjects after calculation was reviewed and the FHVP-CVP gradient was analyzed as a numeric continuous variable instead of a categorical one. Due to the reduced sample size, paired randomization according to the Child-Pugh’s Score was adopted, in order to prevent significant imbalances between study groups regarding liver disease severity. Despite specified in the original protocol, the measurement of recipient IVC pressure for assessment of the renal perfusion pressure during the anhepatic phase was not performed in any case due to unpredicted technical and logistical limitations for safe catheterization of the recipient IVC. In order to identify relevant but transitory renal function changes, measurement of Cr during the planned postoperative observation period was more frequently performed than originally proposed. Finally, after renal function data were already collected, we decided to analyze the results incorporating the RIFLE-AKIN criteria for definition and classification of ARF, which became available in 2004. The authors state that all recorded baseline and outcome measures are included in the present report.

## Results

**Fig 1:**
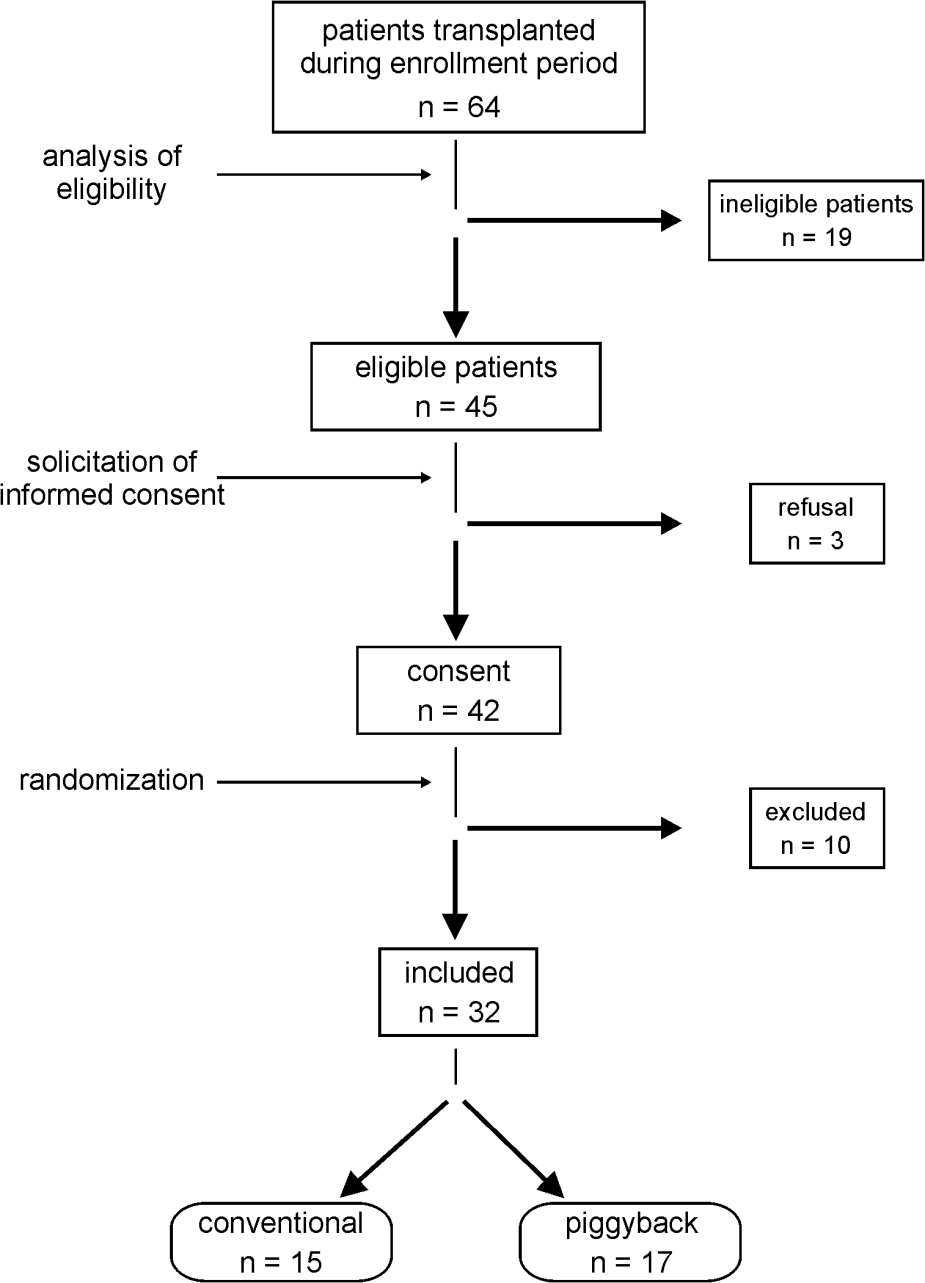
Chart showing the flow of patients during the stages of eligibility, assessment, enrollment and allocation.

Fig 1 summarizes the flow of patients during the stages of eligibility assessment, enrollment and allocation. Sixty-four LTx were performed during the period of enrollment. Of these, nineteen cases did not satisfy the inclusion criteria and were considered ineligible (Table 1). Other three patients refused to participate in the study. The remaining forty-two patients who agreed to participate were randomized immediately before surgery. However, ten cases (5 from conventional and 5 from piggyback group) were excluded after randomization: four because of displacement of the catheter from right hepatic vein (two patients from each surgical group); two, in whom the surgical method predicted by randomization was switched during transplantation (from piggyback to conventional method in both instances); two, who died in the intraoperative period (both from conventional group); one because of hemodynamic instability (piggyback group); and one, due to missing intraoperative data (conventional group). Thus 32 cases were effectively studied, 15 of the conventional and 17 of the piggyback group. The patients’ diagnoses are shown in Table 2.

**Table 1:** Causes of ineligibility among 64 patients transplanted during enrollment period.

Cause of ineligibility		(n = 19)
Age under 18 years		1
Liver retransplantation		3
Acute hepatic failure		5
Living donor transplantation		3
Preferential option by conventional method	Huge hepatomegaly*	2
	TIPS stent dislocation in the right hepatic vein	1
	Budd-Chiari syndrome	1
	Familial amyloidotic polyneuropathy type 1	3

TIPS, transjugular intrahepatic portosystemic shunt

*Includes one Gouchet disease and one polycystic disease cases.

**Table 2:** Patient primary diagnoses.

Diagnosis	Conventional (n = 15)	Piggyback (n = 17)	Total (n = 32)
Hepatitis C	7	7	14
Hepatitis B	1	2	3
Hepatitis C and B	1	0	1
Alcoholic cirrhosis	3	0	3
Hepatitis C and alcohol	0	1	1
Hepatitis C and B and alcohol	0	1	1
Primary sclerosing cholangitis	0	2	2
Primary biliary cirrhosis	0	2	2
Ductopenic disease	1	0	1
Autoimmune hepatitis	1	0	1
Cryptogenic cirrhosis	0	2	2

Completed data were obtained from each included patient. The distribution of donor characteristics is displayed in Table 3. Donor’s demographics, diagnosis, and geographic organ sharing were comparable amongst conventional and piggyback groups. Comparison of recipient baseline characteristics and operative data is shown in Table 4. The two groups present similar gender, age, Child-Pugh and MELD scores, preoperative Cr values, operative time, graft cold ischemia time and intraoperative red packed cells transfusions requirements. All transplants were performed using full sized grafts. Abdominal drains were placed in only one patient.

**Table 3:** Donor characteristics.

		Conventional (n = 15)	Piggyback (n = 17)	p
**Gender**	male/female	8/7	7/10	0.74
**Age** (years)	mean ± SD	35.0 ± 16.0	36.6 ± 13.5	0.76
	median (range)	36.0 (9–60)	40.0 (14–57)	—
**Weight** (kg)	mean ± SD	62.3 ± 13.5	69.2 ± 11.2	0.12
	median (range)	65.0 (30–85)	70.0 (45–85)	—
**Diagnosis**	Stroke	8	8	0.78
	Trauma	6	6	
	Others	1	3	
**Sharing**	Local	13	15	1.00
	Regional	1	1	
	National	1	1	

**Table 4:** Patient baseline characteristics and operative data.

		Conventional (n = 15)	Piggyback (n = 17)	p
**Gender**	male/female	11/4	11/6	0.71
**Age** (years)	mean ± SD	47.8 ± 2.9	46.6 ± 2.7	0.76
	median (range)	47.0 (28–70)	48.0 (18–64)	—
**Child-Pugh score**	A	2	2	1.00
	B	9	11	
	C	4	4	
**MELD score**	mean ± SD	14.3 ± 1.5	14.3 ± 4.2	0.97
	median (range)	12.8 (6.8–24.8)	13.9 (7.6–21.2)	—
**Creatinine** (mg/dL)	mean ± SD	1.01 ± 0.29	1.03 ± 0.49	—
	median (range)	1.0 (0.5–1.5)	0.8 (0.6–2.6)	0.47
**Operative time** (min)	mean ± SD	724.0 ± 115.3	649.3 ± 156.6	0.14
	median (range)	690 (555–925)	600 (435–960)	—
**Graft cold ischemia time** (min)	mean ± SD	555.1 ± 160.9	582.4 ± 172.7	0.65
	median (range)	530 (261–890)	550 (340–900)	—
**Red packed cells use** (units)	mean ± SD	13.9 ± 10.3	9.0 ± 7.6	0.13
	median (range)	9 (4–34)	6 (3–35)	—

**Fig 2:**
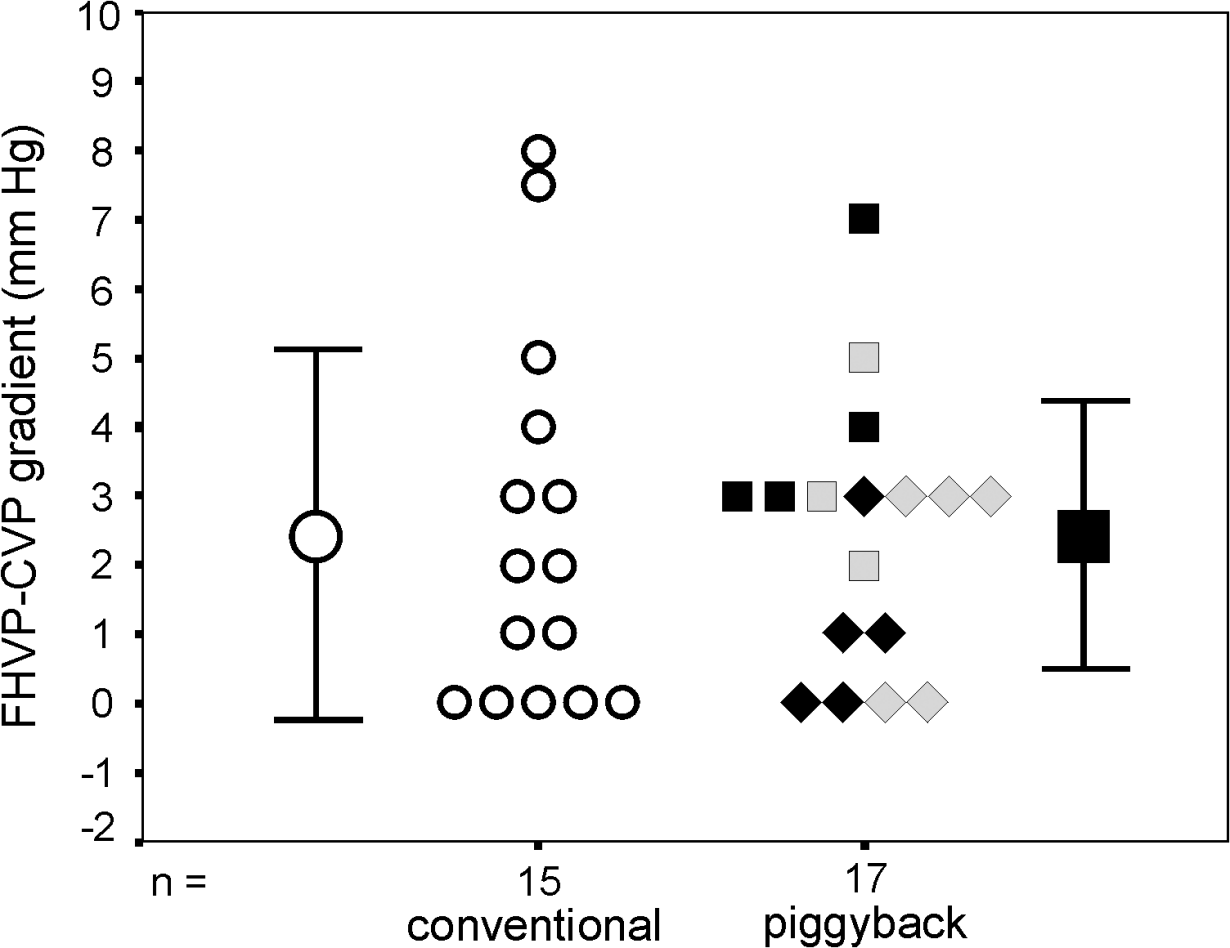
Gradient between the free hepatic vein pressure and the central venous pressure (FHVP-CVP). Large symbols represent mean FHVP-CVP gradient values and bars represent standard deviation. Small symbols represent individual FHVP-CVP gradient values. Median FHVP-CPV gradients are similar in conventional and piggyback techniques (p = 0.74). In the piggyback group, four variants of hepatic venous outflow reconstruction were used: anastomosis to the cuff of the middle and left hepatic veins (grey square); to the right and middle hepatic veins (grey diamond); to the right, middle and left hepatic veins (black diamond); and side-to-side anastomosis between graft’s and recipient’s vena cava (black square). ANOVA comparison showed a significant difference between these 4 reconstructions (p = 0.04).

Hepatic vein and right atrium pressure measurements were performed 174.0 ± 87.1 minutes after graft revascularization, on average. The observed FHVP-CVP gradient values in the conventional and piggyback groups are shown in Fig 2. Mean gradient value was 2.43 ± 2.68 vs. 2.41 ± 1.94 mm Hg, respectively. Median FHVP-CVP gradient value is 2 mm Hg in the conventional group (range 0–8 mm Hg) and 3 mm Hg, in the piggyback group (range 0–7 mm Hg). This difference was not statistically significant (p = 0.73). A FHVP-CVP gradient higher than 3 mm Hg was observed in 26.7% of the cases (4/15) in the conventional and in 17.6% of the cases (3/17) in the piggyback group. There is no statistically significant difference between these rates (p = 0.68).

In the piggyback group, the suprahepatic portion of IVC of the graft was anastomosed with the middle and left hepatic veins (ML) in 3 cases; with the right and middle hepatic veins (RM) in 5 cases; with the right, middle and left hepatic veins (RML) in 5 cases and by means of a SS anastomosis between the vena cava of the graft and of the recipient in 4 cases (Fig 2). A post-hoc ANOVA comparison showed a significant difference between the FHVP-CVP gradient observed in these 4 types of piggyback hepatic venous outflow reconstruction (p = 0.04). However, on Bonferroni’s multiple comparison analysis, no statistically significant paired difference was identified.

There is no statistically significant difference between the conventional (1/15 cases) and the piggyback (2/17 cases) groups regarding massive ascites development (p = 1.00).

**Fig 3:**
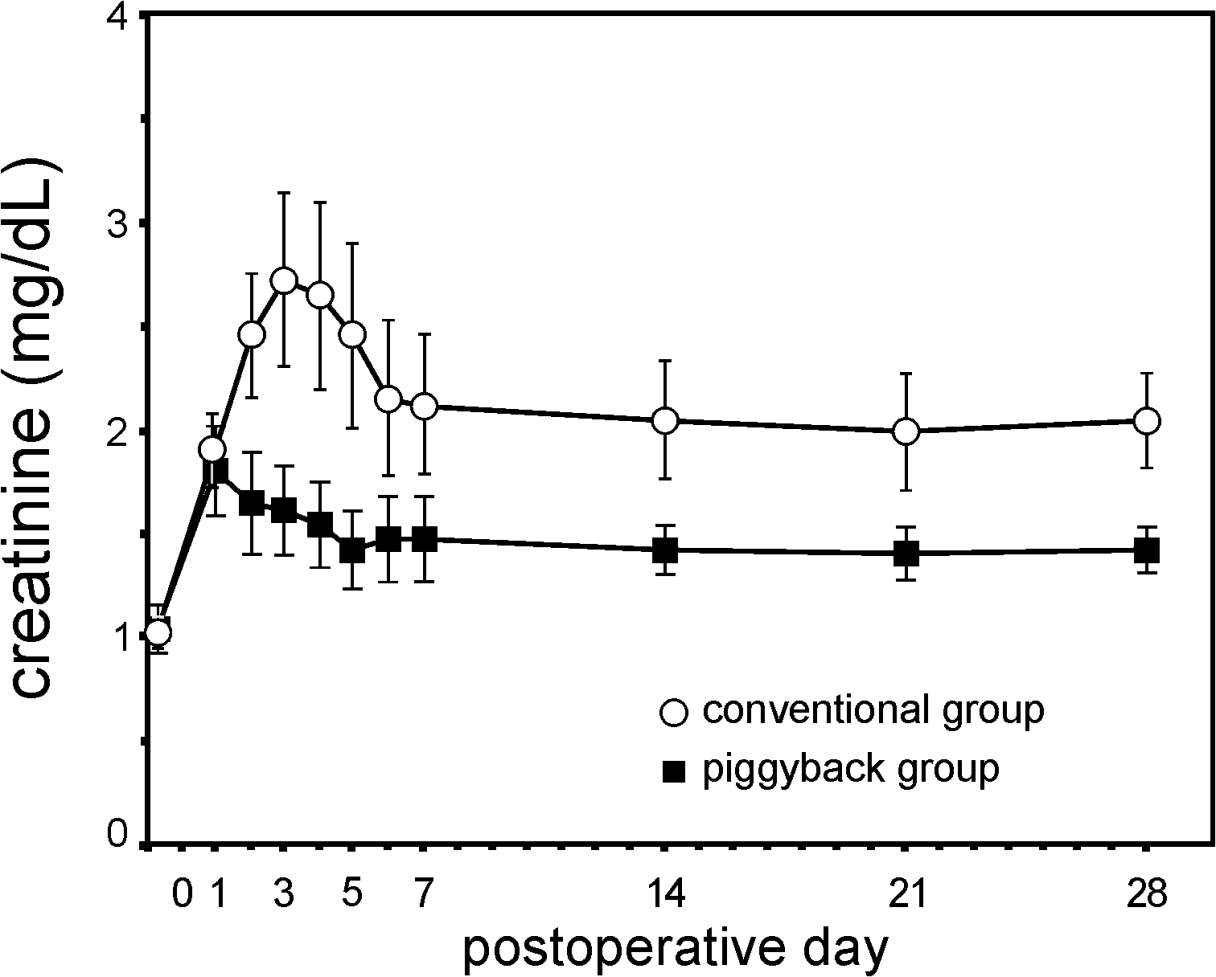
Mean serum creatinine (Cr) values from the preoperative period till the 28^th^ postoperative day. Bars represent standard error. The estimated marginal mean for Cr was significantly higher in conventional than in piggyback group (2.14 ± 0.26 vs. 1.47 ± 0.15 mg/dL; p = 0.02).

**Fig 4:**
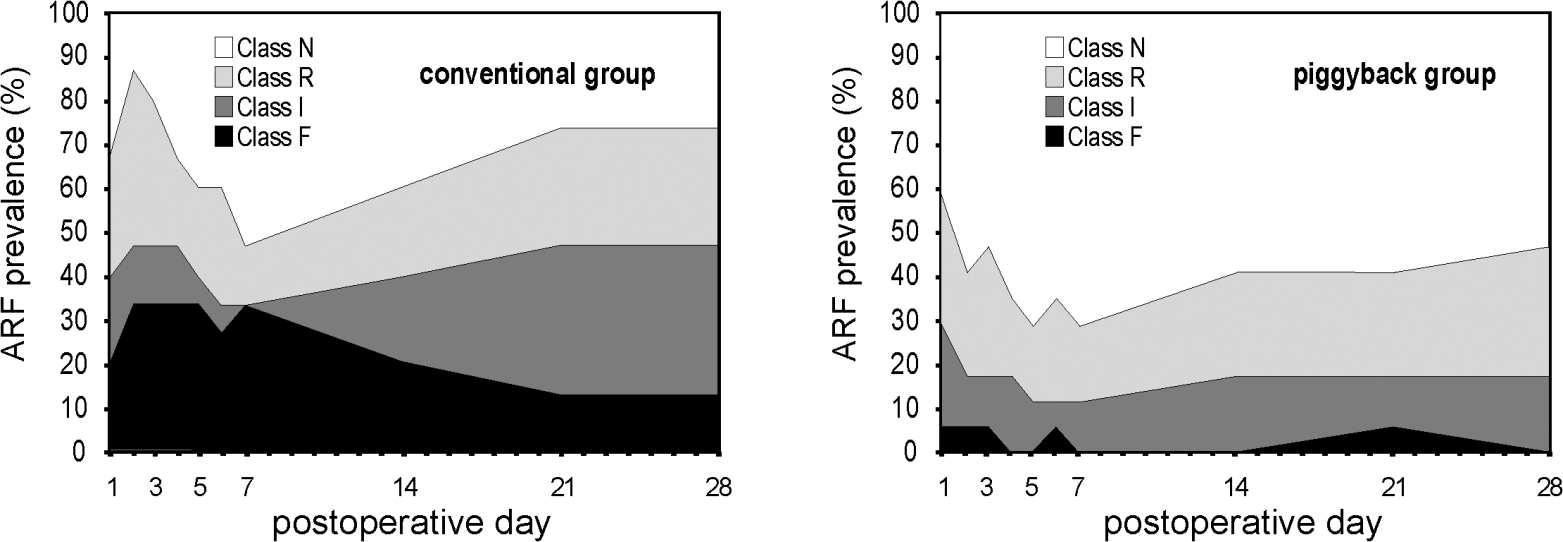
Prevalence of acute renal failure (ARF) till the 28^th^ postoperative day according to RIFLE-AKIN staging. Class N = no renal impairment; Class R = risk; Class I = injury; Class F = failure. The conventional method presented a significantly higher prevalence of severe ARF (Class I + F) during the first 28 postoperative days (OR = 3.207; 95% CI, 1.010 to 10.179; p = 0.048).

Fig 3 shows the distribution of the mean Cr values from the preoperative period till the 28^th^ PO in both groups. Fig 4 shows the prevalence of ARF according to RIFLE-AKIN staging in conventional and piggyback groups up to the 28^th^ PO. Based on the QIC values, GEE analysis of Cr longitudinal measurements was performed assuming exchangeable correlation while a nine-dependent working correlation structure was preferred for ARF assessment. GEE estimated marginal mean for Cr was significantly higher in conventional than in piggyback group (2.14 ± 0.26 vs. 1.47 ± 0.15 mg/dL; p = 0.02). The conventional method presented a significantly higher prevalence of severe ARF on the first 28 PO days (OR = 3.207; 95% CI, 1.010 to 10.179; p = 0.048).

Considering the entire study population, cumulative incidence of overall ARF (RIFLE-AKIN Class R + I + F) was 79% within 48 hours, 86% during the first week after transplantation and 90% at the 28^th^ PO (87%, 87% and 93%, for the conventional group, versus 71%, 78% and 85%, for the piggyback group). At the same time intervals, cumulative incidence of severe ARF (Class I + F) was 41%, 50% and 53%, respectively (53%, 67% and 67%, for the conventional group, versus 29%, 35% and 41%, for the piggyback group). Hemodialysis was required for only one patient, submitted to conventional LTx. In this case, renal replacement therapy was initiated at the 17^th^ PO.

**Fig 5:**
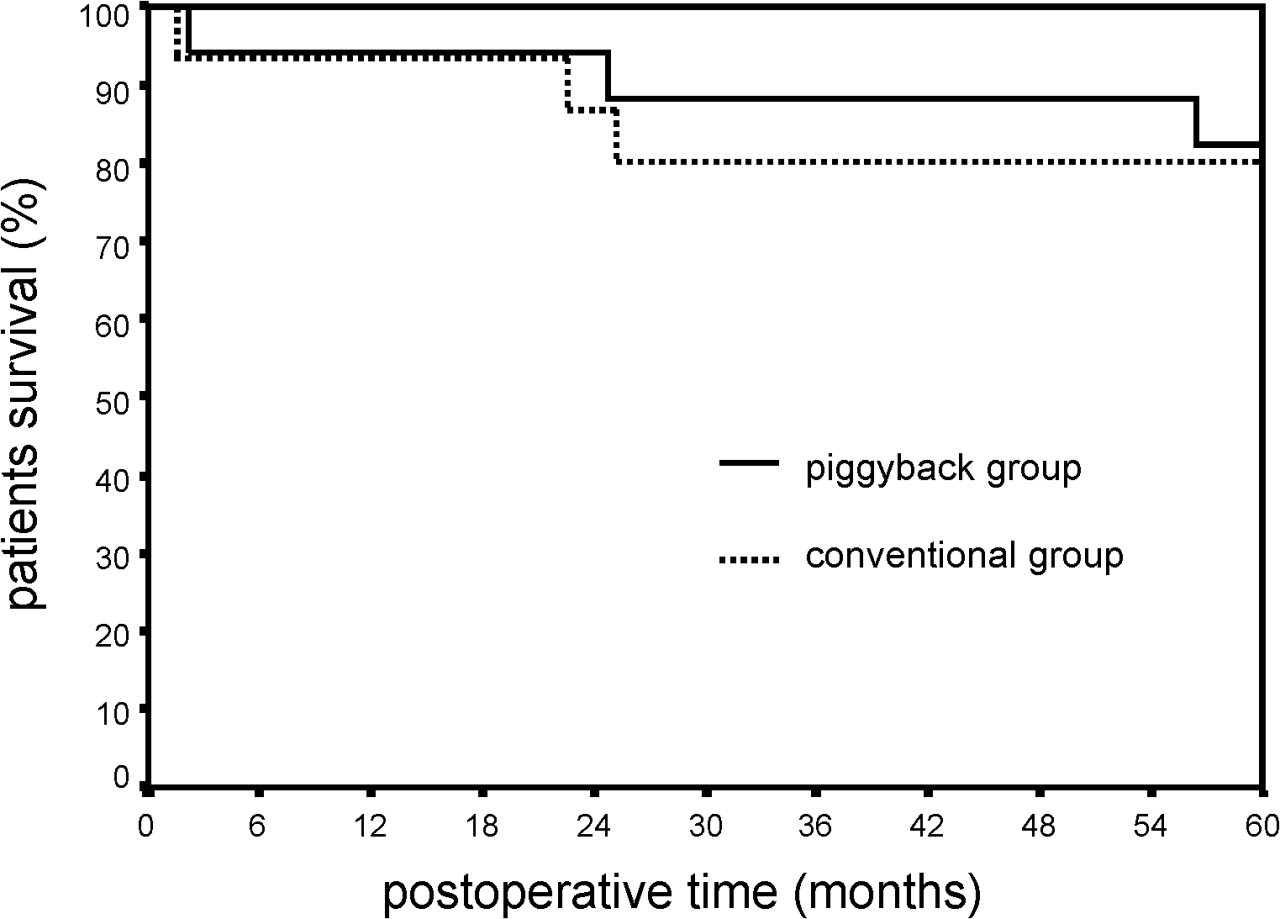
Patient’s postoperative Kaplan-Meir survival curves in conventional and piggyback groups (p = 0.32).

Patient’s postoperative Kaplan-Meir survival curves are shown in Fig 5. One-, 3- and 5-years patients survival was 93%, 80% and 80%, in the conventional group, and 94%, 88% and 82%, in piggyback group, respectively (p = 0.32).

## Discussion

Despite the popularization of piggyback LTx, the benefits of this technique on clinical outcomes over conventional LTx with VVB are yet under debate [4]. Unfortunately, most of the literature concerning the advantages of conventional or piggyback LTx techniques is based on historical comparisons. In 2011, a systematic review on this issue concluded that currently there is no evidence to recommend or refute the use of piggyback LTx [51].

Venous outflow obstruction, caused by either graft rotation or anastomotic stricture, has been a cause for concern after the piggyback technique. To the best of our knowledge, the present study is the first prospective randomized clinical trial designed to compare the hepatic venous outflow between conventional or piggyback LTx. Additionally, postoperative renal function was also compared between the two methods.

According to previously reported studies, hepatic venous outflow was analyzed based on the FHVP-CVP gradient [17,25]. We found very similar FHVP-CVP gradients in conventional and piggyback groups. Frequency of venous outflow blockade, defined by the FHVP-CVP gradient > 3 mm Hg, was not statistically different between both methods and there was no statistically significant difference regarding massive ascites development.

Our findings differ from the results of a retrospective study performed by Cirera et al. that reported a three times higher incidence of massive ascites after piggyback than in conventional LTx [25]. Considering the entire group, they found a significantly higher FHVP-CVP gradient in patients with ascites than in those without this complication (10.4 ± 3.7 vs. 5.6 ± 4.6 mm Hg; p<0.01). It is important to note that the mean gradient in the group without ascites was higher than 3 mm Hg. It means that several patients who presented a gradient considered hemodynamically significant did not develop clinical manifestations. We observed a similar pattern in the present study: among the 7 patients with hemodynamic hepatic venous block criteria, only 2 developed massive ascites. This discrepancy suggests that consideration should be given on adopting a threshold higher than 3 mm Hg for characterizing a clinically relevant FHVP-CVP gradient [52].

The risk of upper caval outflow compromise after piggyback LTx may be strongly related to technical aspects of venous reconstruction. Several different methods of piggyback upper vena cava anastomosis have been reported in the literature and, currently, a standard international operative technique does not exist [5]. The greater venous drainage disorder observed by Cirera et al. in the piggyback method may be related to the exclusive use of the ML anastomosis in their study [25]. This variant, which was extensively employed in the early descriptions of piggyback LTx, may have an increased likelihood of kinking or stenosis due to the long length of the common stump of the middle and left hepatic veins, the spatial orientation of their axis to the left and the narrow outflow orifice in IVC [15,16,21,24]. In fact, after analysis of their results, the authors attempt to reduce the risk of hepatic venous outflow obstruction by using RML anastomosis and, since then, the occurrence of ascites after piggyback LTx became anecdotal in their service. Similarly, most centers performing piggyback LTx have extensively replaced ML anastomosis for wider, shorter and better positioned venous outflow alternatives, like RML and SS [5,15,16,17,53].

In the present study, four variants of hepatic venous outflow reconstruction were used in the 17 cases of the piggyback group. RML, RM and SS anastomosis were widely preferred and ML reconstruction was used in only three cases (17.7%). We believe that the similar venous outflow hemodynamic profile observed between conventional and piggyback LTx in our study reflects the reduced frequency of hepatic outflow block with the current techniques of piggyback venous reconstruction.

Conversely, it should be realized that avoiding cross-clamping of IVC throughout LTx is one of the most appealing aspects of IVC preservation. Widening the piggyback outflow reconstruction may impair to varying degrees the venous return through the recipient IVC, which might reduce the hemodynamic advantages achieved by maintaining an unobstructed caval flow during the anhepatic phase. In effect, while in ML variant clamping of the IVC was minimal, RML and SS techniques involves partial side clamping of the recipient IVC which can produce transient reductions in IVC blood flow and cardiac output and lead to some renal congestion [54]. Unfortunately, we were unable to perform serial measurements of the renal perfusion pressure during the anhepatic phase in the present study despite prespecified in our original protocol.

The RIFLE-AKIN criteria allow early detection of ARF in mildly affected patients, in whom kidney injury can be prevented (“Risk” stratum or Class R), but limits the specificity for true renal dysfunction at this stage. Therefore, we decided to perform statistical analysis combining Class I with Class F patients, as a single group with more markedly affected renal function, like other authors did [55,56].

Using the RIFLE-AKIN classification, we found a relatively high cumulative incidence of overall (90%) and severe (53%) ARF, four weeks after LTx. Using the same standardized diagnostic criteria, the incidence of ARF after LTx differs quite widely among the literature, ranging from 31% to 93%, for overall ARF, while severe ARF (Class I + F) is reported in 10% to 64% of the recipients [31,55,57–65]. Such disparities may be partially explained by different characteristics of the population being studied or by the discrepant time points used for assessment of ARF staging, varying from 48 hours to more than 30 PO days. In the present study, we observed an increment higher than 10% on cumulative incidence of overall and severe ARF from 48 hours to the 28^th^ PO.

It has been demonstrated that, in respect to Class N, the risk of poor clinical outcomes almost linearly increases with each worse RIFLE-AKIN stage [65,66]. Despite the high rate of overall and severe ARF found in the current study, our patients experienced a low mortality, with one- and five-years survival rates above 90% and 80%, respectively. We believe that these satisfactory short- and long-term outcomes may result from the interaction between patient’s selection and specific perioperative anesthetic, surgical and intensive care practices.

The most relevant finding in our study was a significant worse postoperative renal function following conventional with VVB than piggyback LTx. These results are in agreement to a previous prospective randomized study, reported by Jovine et al. [67], that found a significant higher probability of ARF (defined as Cr higher than or equal to 2.5 mg/dL) after conventional with VVB than piggyback LTx (30.8% vs. zero; p<0.05). It’s important to note that the delineation of both trials does not allow attributing the development of postoperative ARF to the type of hepatic venous outflow reconstruction by itself (conventional versus piggyback), otherwise to the distinct procedure used in each surgical group for sustaining venous return during anhepatic phase (i.e. VVB versus caval flow preservation).

Caval flow preservation and VVB are different strategies to reduce systemic hemodynamic derangements and renal venous congestion, which are considered leading intraoperative factors contributing to renal dysfunction after LTx. As expected, piggyback technique significantly reduced the risk of RIFLE-AKIN overall ARF up to 5 days after LTx compared to full caval clamping (conventional without VVB) in a prospective observational study of 59 patients [59]. In contrast, in a prospective randomized clinical trial, the use of VVB in conventional LTx did not decrease postoperative renal impairment, despite improving systemic and renal hemodynamics during full caval cross-clamping [68]. Sakai et al. retrospectively compared three surgical groups: 174 patients submitted to piggyback LTx, 148 patients transplanted using piggyback with VVB and 104 patients submitted to conventional LTx with VVB [69]. They found a significant lower incidence of postoperative Class F ARF using caval flow preservation when compared to VVB, irrespective of graft implantation by conventional or piggyback method. The authors concluded that the benefit of piggyback technique was decreased when it was combined with VVB. Taken together, these results suggest that, for reasons not fully understood, VVB does not prevent renal damage associated to IVC cross-clamping and even may has a detrimental effect to the kidney. Considering that currently less than 25% of the liver transplant centers always use VVB when not preserving IVC [5], could be interesting to conduct further prospective randomized studies comparing ARF after piggyback or conventional LTx without VVB.

Prior to conclude, it is important to consider some weakness of the present study. First of all, this trial was mainly designed as a hemodynamic investigation and was powered to identify small differences in regard to early hepatic venous pressure gradients after LTx, which can be considered a very sensitive approach for detection of systematic venous outflow changes. However, considering the small probability of persistent clinically detectable outflow block with current surgical techniques, usually below 2%, a much larger sample size would be required in order to distinguish slight disparities between intervention groups regarding some relevant clinical variables that were not originally considered primary end points of the present study. For this reason, the lack of a significant difference in relation to clinical variables, such as massive ascites, should be suspiciously interpreted. Secondly, the method of venous reconstruction used in the piggyback group was not standardized. Although some evidence has emerged from our results, suggesting distinct venous outflow profile according to each variant of piggyback caval implantation, we are unable to explore this finding. Further prospective studies comparing different hepatic venous anastomotic techniques in piggyback LTx are desirable to verify these data. Thirdly, according to the original design of our trial, outcome data were not prospectively collect from 10 patients who deviated from protocol after randomization. For this reason, we are unable to perform an intention-to-treat analysis. In spite of this, the exclusion of these cases did not produce any apparent imbalance between the two study groups. Lastly, we cannot ignore the fact that our study population was constituted more than a decade ago. For this reason, some factors that can influence the risk of either postoperative ARF or mortality, like blood transfusion requirements and mean MELD score [62], were substantially different in our patients than practiced nowadays [70]. However, although the probability of a historical bias could be deeply considered in a retrospective study, this concern is less relevant in a prospective randomized trial. In fact, the main principle of this approach is to compare groups without any apparent imbalances in order to indentify and isolate the influence of the intervention of interest on the selected outcome variables. In the present trial, the randomization process succeed producing independent conventional and piggyback groups that are proved to be quite similar regarding all baseline and operative variables analyzed.

Regardless of these limitations, by confirming a lower prevalence of severe ARF after piggyback LTx, we believe that the present randomized prospective trial provides a methodologically robust evidence of a relevant clinical benefit of this method over the conventional with VVB technique. Therefore, the data presented support a recommendation for routine use of piggyback method avoiding VVB.

## Conclusions

It can be concluded that patients submitted to LTx using conventional or piggyback methods present similar results regarding venous outflow drainage of the graft. Renal function is significantly worse after conventional with VVB transplantation.

## Supporting Information

S1 CONSORT Checklist CONSORT checklist. (PDF)

S1 Protocol Portuguese language hepatic venous hemodynamic protocol. (PDF)

S2 Protocol English version hepatic venous hemodynamic protocol. (PDF)

S3 Protocol Portuguese language renal function protocol. (PDF)

S4 Protocol English version renal function protocol. (PDF)
