# Supplementary material for: Prospective Randomized Trial Comparing Hepatic Venous Outflow and Renal Function after Conventional versus Piggyback Liver Transplantation
Source: PLoS One. 2015 Jun 26;10(6):e0129923. doi: 10.1371/journal.pone.0129923 (PMC4482688; doi:10.1371/journal.pone.0129923)
Supplement: S1 Protocol — (PDF) [file pone.0129923.s002.pdf]

PROJETO DE PESQUISA

COMPARAÇÃO DA FREQÜÊNCIA DE BLOQUEIO DA DRENAGEM VENOSA EM FÍGADOS TRANSPLANTADOS PELOS MÉTODOS CONVENCIONAL OU *PIGGYBACK*

Pesquisador Responsável: Dr. Paulo Celso Bosco Massarollo

PROJETO DE PESQUISA

COMPARAÇÃO DA FREQÜÊNCIA DE BLOQUEIO DA DRENAGEM VENOSA EM FÍGADOS TRANSPLANTADOS PELOS MÉTODOS CONVENCIONAL OU *PIGGYBACK*

1. Introdução e justificativa

Um aspecto técnico de grande importância no transplante de fígado (Tx), é a manutenção do estado hemodinâmico do receptor durante a fase anepática. Na técnica dita *convencional,* o fígado é retirado em bloco junto com a porção retro-hepática da veia cava inferior, interrompendo o retorno venoso da porção infra-diafragmática. Para evitar as conseqüências desta manobra, utiliza-se uma derivação veno-venosa temporária pela qual o sangue proveniente da veia cava inferior e da veia porta é devolvido no território da veia cava superior, por meio de um uma bomba centrífuga de circulação extra corpórea.[1]

Mais recentemente, descreveu-se a realização do Tx pelo método de *piggyback*, onde o fígado doente é retirado com preservação da porção retro-hepática da veia cava inferior.[2] Desta forma, o fluxo pela veia cava inferior pode ser mantido, eliminando-se a necessidade de derivação veno-venosa. Este método evita os riscos da circulação extra-corpórea como a embolia gasosa, o tromboembolismo pulmonar[3] e a formação de fístulas linfáticas nas regiões axilar e inguinal,[4] onde são realizados incisões para colocação dos cateteres. Adicionalmente, existe uma redução dos custos do procedimento (cateteres específicos de derivação venosa, bomba de circulação extra corpórea, perfusionista, etc).

A essas vantagens, contrapõem-se a suspeita de uma maior incidência de bloqueio da drenagem venosa do fígado em pacientes operados pelo método *piggyback*, o que tem sido relatado por alguns autores[5] e negado por outros.[3,6,7] Apesar da controvérsia, desconhece-se qualquer estudo prospectivo e randomizado que procurou comparar a freqüência desta complicação nos dois métodos.

2. Objetivo

O objetivo desta pesquisa é comparar a freqüência de bloqueio da drenagem venosa do fígado em Tx realizados pelo método convencional ou *piggyback*.

3. Casuística e Método

Serão estudados prospectivamente os 42 pacientes candidatos a Tx que participarão dos protocolos de pesquisa "Avaliação de citoquinas inflamatórias no transplante de fígado realizado por dois métodos operatórios: convencional e piggyback" e "Avaliação da translocação bacteriana no transplante de fígado", e que concordarem em também participar desta pesquisa. Estes projetos, que já foram analisados e aprovados pelo Departamento de Cirurgia da FMUSP e pela CAPPesq, prevêem a randomização de pacientes de ambos os sexos e com idade entre 18 e 60 anos, em dois grupos: Tx pelo método convencional e Tx pelo método *piggyback.* A randomização será realizada imediatamente antes da cirurgia, por meio de tabela de números equiprováveis. Os protocolos definem como critérios de exclusão a "polineuropatia amiloidótica familiar", o retransplante de fígado, a presença de infecção ativa, a impossibilidade técnica de realização do método operatório previsto após a randomização, a necessidade de realização de anastomose porto-cava temporária e os casos de fígado reduzido. Como a casuística deste protocolo será a mesma, serão adotados os mesmos critérios.

Será avaliado, nos dois grupos, o gradiente de pressão entre a veia hepática e o átrio direito. Todos os procedimentos necessários para a obtenção destas medidas constam nos protocolos já aprovados ou são realizados rotineiramente no Tx. Esses protocolos prevêem a introdução de um catéter na veia hepática do enxerto, para coleta de amostras de sangue. Na presente pesquisa, esta via será utilizada para medida da pressão da veia hepática. Já a pressão do átrio direito é registrada continuamente, em todo Tx, por meio de cateter de Swan-Ganz (procedimento de rotina).

A medida das pressões da veia hepática e do átrio direito será feita uma única vez, junto com as últimas coletas dos demais protocolos, ou seja, 120 minutos após a revascularização do fígado. Será considerado "bloqueio venoso hepático" a existência de gradiente pressórico igual ou superior a 3mmHg.[6]

A freqüência de "bloqueio venoso" será comparada, entre os dois grupos, por meio do teste χ^2 de Pearson com correção de Yates. Será considerado nível de significância de 5%. O tamanho previsto da amostra (21 pacientes em cada grupo) é suficiente, caso a freqüência de bloqueio seja 0%, no grupo convencional, e 25%, no grupo *piggyback*. Estas proporções

foram escolhidas considerando que a ocorrência de complicações da drenagem venosa é muito rara no transplante convencional enquanto, no *piggybac*k, existem relatos de uma incidência de até 25%.[6]

6. Referências bibliográficas

1. Shaw, B; Martin, D; Marquez,J. – Venous bypass clinical liver transplantation. *Ann Surg.* 1984; 200:524-9.

2. Tzakis, A; Todo, S; Starzl, T. – Orthotopic liver transplantation with preservation of the inferior vena cava. *Ann Surg.*1989; 210:649-55.

3. Fleitas, MG; Casanova, D; Martino, E; Maestre, JM; Herrera, L; Hernanz, F; Rabanal, JM; Pulgar, S; Solares, G. – Could the piggyback operation in liver transplantation be routinely used ? *Arch Surg.* 1994;129:842-5.

4. Stieber, AC. – One surgeon's experience with the piggyback versus the standard technique in orthotopic liver transplantation: is one better than the other ? *Hepato-Gastroenterology.*1995; 42:403-5.

5. Stieber, AC; Gordon, RD; Bassi, N. – A simple solution to a technical complication in "piggyback" liver transplantation. *Transplantation.*1997; 64:454-5.

6. Ducerf, C; Rode, A; Adham, M; Roche, E; Bizollon, T; Baulieux,J ; Pouyet, M. – Hepatic outflow study after piggyback liver transplantation. *Surgery.*1996; 120:484-7.

7. Lauchart, W; Köveker, G; Viebahn, R; Schott, U; Judt-Stelzer,G.- No impairment of hepatic venous outflow after "piggyback" liver transplantation. *Transplantation Proceedings,*1997; 29:2864-5.

PROTOCOLO DE PESQUISA:

Comparação da freqüência de bloqueio da drenagem venosa em fígados transplantados pelos métodos convencional ou *piggyback*

Identificação: ____

Nome:____

Idade: ______ **RGHC**: ______________________ **Caso n º**: __________

Data do Tx: ____/____/____

Indicação do Tx: ____

Tipo de Cirurgia : ( ) Convencional

( ) *Piggyback*

Critério de Inclusão:

Consentimento pós-informação assinado Sim ( ) Não ( )

Idade maior ou igual a 18 anos Sim ( ) Não ( )

Idade inferior ou igual a 60 anos Sim ( ) Não ( )

Critérios de Exclusão:

Polineuropatia amiloidótica familiar Sim ( ) Não ( )

Retransplante de fígado Sim ( ) Não ( )

Infecção ativa Sim ( ) Não ( )

Impossibilidade de aplicação do método previsto Sim ( ) Não ( )

Anastomose porto-cava Sim ( ) Não ( )

Fígado reduzido Sim ( ) Não ( )

Obs: Serão estudados os pacientes com resposta “sim” a todos os critérios de inclusão e “não” a todos os critérios de exclusão.

Resultado:

Pressão de veia hepática livre: ______mmHg

Pressão venosa central: ______ mmHg

Gradiente de pressão: ______ mmHg
