# Supplementary material for: Prospective Randomized Trial Comparing Hepatic Venous Outflow and Renal Function after Conventional versus Piggyback Liver Transplantation
Source: PLoS One. 2015 Jun 26;10(6):e0129923. doi: 10.1371/journal.pone.0129923 (PMC4482688; doi:10.1371/journal.pone.0129923)
Supplement: S2 Protocol — (PDF) [file pone.0129923.s003.pdf]

RESEARCH PROTOCOL

Comparison of the frequency of venous outflow blockade in liver grafts transplanted using conventional or piggyback method

Responsible investigator: Dr. Paulo Celso Bosco Massarollo

1- Introduction and justification

A technical aspect of great importance in liver transplantation (LTx) is the maintenance of the hemodynamic status of the recipient during the anhepatic phase. In the conventional technique, the liver is removed *en bloc* with the retrohepatic portion of the inferior vena cava (IVC), interrupting the venous return of the infradiaphragmatic bed. In order to overcome the consequences of this maneuver, a temporary venovenous bypass (VVB) driven by a centrifugal pump is usually used, allowing diversion of the IVC blood flow to the superior vena cava.(1) More recently, the piggyback method of LTx was described, in which the diseased liver is removed with preservation of the retrohepatic portion of the IVC.(2) Thus, flow through IVC can be maintained, precluding the need for VVB and its inconveniences.(6) This methods avoid the risk of extracorporeal circulation such as air and thrombotic pulmonary embolism,(3) and lymphatic fistulas in the axillary and inguinal regions,(4) where incisions for the placement of catheters are performed. In addition, there a lower cost of the procedure (specific catheters, extracorporeal pump machine, equipment operator, and so on).

In contrast to these advantages, there is a concern regarding a higher incidence of hepatic venous outflow blockade in piggyback LTx recipients, which has been

reported by some authors[5] and denied by others.[3,6,7] Notwithstanding this controversy, we are unaware of any prospective randomized trial that it looked to compare the frequency of this complication in the two methods.

2- Aim

To compare the frequency of hepatic venous outflow blockade after conventional VVB or piggyback LTx.

3- Patients and methods

Forty-two LTx candidates, who will participate of the research protocols ***"Evaluation of inflammatory cytokines in two operative methods of liver transplantation: conventional or piggyback"*** and ***"Evaluation of bacterial translocation in liver transplantation"***, and that agree to also participating of this research, will be prospectively studied. These protocols, which have been already analyzed and approved by the Department of Surgery of FMUSP (Faculdade de Medicina da Universidade de São Paulo) and by CAPPesq (Ethics Committee of the Institution - Comissão de Ética para Análise de Projetos de Pesquisa do Hospital das Clínicas da Faculdade de Medicina da Universidade de São Paulo), admitted randomization of both genders patients, with age between 18 and 60 years, to two groups: LTx performed using the conventional or the piggyback method. Randomization will be conducted immediately before the surgery, by using a table of random numbers.

Exclusion criteria include “familial amyloidotic polyneuropathy” diagnosis, liver retransplantation, active infection, technical impossibility of accomplishment of the surgical method predicted by randomization, necessity of temporary porto-caval anatomosis, and reduced (partial) liver graft. As the patients will be shared with these two studies, the same criteria will be adopted in the current protocol.

In the two groups, the pressure gradient between the hepatic vein and the right atrium will be evaluated. All the necessary procedures for these measurements are also required in the two previously approved protocols or are routinely performed in LTx. According to these protocols, a catheter will be introduced into the graft’s right hepatic vein for blood samples collection. In the current research, this catheter will be used for free hepatic vein pressure measurement. Right atrium pressure was obtained in all LTx by using a Swan-Ganz catheter (routine procedure).

Measurement of hepatic vein and right atrium pressure will be performed once, simultaneously with the last blood sample collection required for the other research protocols, it means, 120 minutes after graft revascularization. “Hepatic venous outflow block” will be considered when a FHVP-CVP gradient higher than 3 mm Hg is registered.[6]

The frequency of “hepatic venous outflow block” will be compared between the two groups by Yates-corrected Pearson’s chi-squared test. A 5% significance level will be considered. The sample size (21 patients in each group) would be sufficient expecting an outflow block frequency of 0% and 25% in conventional and piggyback groups, respectively. These ratios were selected considering that the occurrence of

complications of the venous drainage are very rare in conventional transplantation while an incidence as higher as 25% has been reported after piggyback.[(6)]

References

1. Shaw, B; Martin, D; Marquez,J. – Venous bypass clinical liver transplantation. *Ann Surg.* 1984; 200:524-9.

2. Tzakis, A; Todo, S; Starzl, T. – Orthotopic liver transplantation with preservation of the inferior vena cava. *Ann Surg.*1989; 210:649-55.

3. Fleitas, MG; Casanova, D; Martino, E; Maestre, JM; Herrera, L; Hernanz, F; Rabanal, JM; Pulgar, S; Solares, G. – Could the piggyback operation in liver transplantation be routinely used ? *Arch Surg.* 1994;129:842-5.

4. Stieber, AC. – One surgeon's experience with the piggyback versus the standard technique in orthotopic liver transplantation: is one better than the other ? *Hepato-Gastroenterology.*1995; 42:403-5.

5. Stieber, AC; Gordon, RD; Bassi, N. – A simple solution to a technical complication in "piggyback" liver transplantation. *Transplantation.*1997; 64:454-5.

6. Ducerf, C; Rode, A; Adham, M; Roche, E; Bizollon, T; Baulieux,J ; Pouyet, M. – Hepatic outflow study after piggyback liver transplantation. *Surgery*.1996; 120:484-7.

7. Lauchart, W; Köveker, G; Viebahn, R; Schott, U; Judt-Stelzer,G.- No impairment of hepatic venous outflow after "piggyback" liver transplantation. *Transplantation Proceedings,*1997; 29:2864-5.
