# Supplementary material for: Prospective Randomized Trial Comparing Hepatic Venous Outflow and Renal Function after Conventional versus Piggyback Liver Transplantation
Source: PLoS One. 2015 Jun 26;10(6):e0129923. doi: 10.1371/journal.pone.0129923 (PMC4482688; doi:10.1371/journal.pone.0129923)
Supplement: S3 Protocol — (PDF) [file pone.0129923.s004.pdf]

PROJETO DE PESQUISA

HEMODINÂMICA SISTÊMICA, PRESSÃO DE PERFUSÃO RENAL E FUNÇÃO RENAL NO TRANSPLANTE DE FÍGADO REALIZADO PELOS MÉTODOS CONVENCIONAL OU *PIGGYBACK*

Pesquisador Responsável: Dr. Paulo Celso Bosco Massarollo

PROJETO DE PESQUISA:

HEMODINÂMICA SISTÊMICA, PRESSÃO DE PERFUSÃO RENAL E FUNÇÃO RENAL NO TRANSPLANTE DE FÍGADO REALIZADO PELOS MÉTODOS CONVENCIONAL OU *PIGGYBACK*

1- Introdução e justificativa

Na técnica convencional do transplante de fígado (Tx), o órgão do receptor é ressecado junto com a porção retro-hepática da veia cava inferior (VCI), que permanece pinçada abaixo do diafragma e acima das veias renais, durante toda a fase anepática. A interrupção do retorno venoso do hemicorpo inferior determina queda da pressão arterial e congestão das veias renais, reduzindo severamente a pressão de perfusão renal (PPR).[1] Para evitar estas alterações hemodinâmicas, descreveu-se a utilização de uma derivação veno-venosa extra-corpórea temporária (*bypass*) pela qual o sangue proveniente da VCI e da veia porta é devolvido no território da veia cava superior.[2] Certamente, esta manobra foi um dos grandes avanços técnicos do Tx, permitindo reduzir a incidência de insuficiência renal no período pós-operatório.[2]

Apesar dessas vantagens, o *bypass* apresenta riscos como a embolia gasosa, o tromboembolismo pulmonar[1,3] e a formação de fístulas linfáticas nas regiões axilar e inguinal,[4] onde são realizados incisões para colocação dos cateteres. Além disso, o custo do procedimento é elevado, devido à necessidade de cateteres específicos, de uma bomba centrífuga especial e de um perfusionista para operar o equipamento. Mais recentemente, descreveu-se a realização do Tx pelo método de *piggyback*, onde o fígado doente é retirado com preservação da porção retro-hepática da VCI.[5] Desta forma, o fluxo pela veia cava inferior pode ser mantido, eliminando-se a necessidade de derivação veno-venosa e seus inconvenientes.[6]

Embora os dois métodos pretendam evitar as conseqüências hemodinâmicas do pinçamento da VCI, este objetivo é alcançado apenas parcialmente em ambos. No Tx com derivação veno-venosa, existe correlação inversa entre o fluxo da circulação extra-corpórea e a PPR.[7] Assim, nos casos com fluxo baixo da derivação, o procedimento pode ser

ineficiente.[8] Já nos Tx realizados pelo método *piggyback*, o pinçamento das veias hepáticas pode reduzir lateralmente a luz da VCI, comprometendo parcialmente o retorno venoso. De fato, descreve-se elevação da pressão da veia cava inferior (PVIC) durante a fase anepática no Tx *piggyback*.[9] Entretanto, no *piggyback*, apesar da elevação da PVCI, a PPR se mantém, devido à elevação concomitante da pressão arterial média (PAM).[9] Desconhece-se qualquer estudo prospectivo e randomizado que procurou comparar os dois métodos em relação à eficiência na descompressão da VCI e às conseqüências hemodinâmicas e renais da redução parcial do retorno venoso.

2- Objetivo

Comparar as alterações da hemodinâmica sistêmica, a pressão de perfusão renal e a função renal pós-operatória em pacientes submetidos a transplante de fígado pelos métodos convencional com derivação veno-venosa ou *piggyback*.

3- Casuística e método

Serão estudados prospectivamente os 42 pacientes candidatos a Tx que participarão dos protocolos de pesquisa "Avaliação de citoquinas inflamatórias no transplante de fígado realizado por dois métodos operatórios: convencional e *piggyback*" e "Avaliação da translocação bacteriana no transplante de fígado", e que concordarem em também participar desta pesquisa. Estes projetos, que já foram analisados e aprovados pelo Departamento de Cirurgia da FMUSP e pela CAPPesq, prevêem a randomização de pacientes de ambos os sexos e com idade entre 18 e 60 anos, em dois grupos: Tx pelo método convencional e Tx pelo método *piggyback.* A randomização será realizada imediatamente antes da cirurgia, por meio de tabela de números equiprováveis. Os protocolos definem como critérios de exclusão a "polineuropatia amiloidótica familiar", o retransplante de fígado, a presença de infecção ativa, a impossibilidade técnica de realização do método operatório previsto na randomização, a necessidade de realização de anastomose porto-cava temporária e os casos de fígado reduzido. Como a casuística deste protocolo será a mesma, serão adotados os mesmos critérios.

Todos os pacientes submetidos a Tx são monitorizados hemodinamicamente por meio de cateter de Swan-Ganz (procedimento de rotina). Este cateter será utilizado para

medida do débito cardíaco e da pressão venosa central. A PAM é registrada continuamente em todos os casos (procedimentos de rotina). Os valores do débito cardíaco, da pressão venosa central e da PAM serão utilizados para o cálculo da resistência vascular periférica. As avaliações serão realizadas no intra-operatório, em 3 momentos: imediatamente antes da hepatectomia (fase 1); durante a fase anepática (fase 2); uma hora após a reperfusão (fase 3).

A veia femoral esquerda será cateterizada por punção percutânea, pela técnica de Seldinger. Será introduzido um cateter angiográfico de calibre 7 French, com orifício lateral, que será posicionado na VCI, junto à desembocadura das veias renais. O posicionamento do cateter será realizado pelo cirurgião, por palpação. Este cateter será utilizado para medida da PVCI. A PPR será avaliada pela diferença entre a PAM e a PVCI. A medida será realizada nos mesmos momentos da avaliação da hemodinâmica sistêmica.

A avaliação da função renal nos períodos pré e pós-operatório será realizada pela dosagem de uréia e creatinina e pelo cálculo da depuração de creatinina em diurese de 24 horas. Esta avaliação será realizada no pré-operatório num intervalo inferior a 2 meses da realização do Tx. No pós-operatório, a avaliação será repetida no 1º, 5º e 30º dias. Durante a fase anepática, será registrado o débito urinário em ml/kg/minuto.

Os resultados numéricos serão submetidos ao teste de Bartlett para avaliar a homogeneidade das variâncias. Se as variâncias forem uniformes, os grupos serão comparados por meio de análise de variância (ANOVA). Se isto não ocorrer, será utilizado o teste de Kruskal-Wallis. O tamanho da amostra é suficiente para demonstrar uma diferença igual ou superior a 3,08mmHg, entre os valores médios da PPR na fase anepática nos dois grupos, fixando-se os erros do tipo I e II em 5% ($\alpha=\beta=0{,}05$) e estimando-se o desvio padrão da amostra em 2,7mmHg.[8]

4- Referências biliográficas

1. Griffith,BP; Shaw,BWJr; Hardesty,RL;Iwatsuki,S; Bahnson,HT;Starzl,TE. – Veno-venous bypass without systemic anticoagulation for transplantation of human liver – *Surg Gynecol Obstet* 1985, 160: 271-72.

2. Shaw, B; Martin, D; Marquez,J. – Venous bypass clinical liver transplantation. *Ann Surg*. 1984; 200:524-9.

3. Fleitas, MG; Casanova, D; Martino, E; Maestre, JM; Herrera, L; Hernanz, F; Rabanal, JM; Pulgar, S; Solares, G. – Could the piggyback operation in liver transplantation be routinely used ? *Arch Surg.* 1994;129:842-5.

4. Stieber, AC. – One surgeon's experience with the piggyback versus the standard technique in orthotopic liver transplantation: is one better than the other? *Hepato-Gastroenterology.*1995; 42:403-5.

5. Tzakis, A; Todo, S; Starzl, T. – Orthotopic liver transplantation with preservation of the inferior vena cava. *Ann Surg.*1989; 210:649-55.

6. Belghiti, J; Panis, Y; Sauvanet, A; Gayet, B; Fekete, F. – A new technique of caval anastomosis during orthotopic liver transplantation without inferior vena cava occlusion. *Surg Gynecol Obstet* 1992; 175:270-72.

7. Scherer,RU; Giebler,RM; Schmutzler,MJ;Erhard,J; Lange,R; Kox,WJ. – Shuntflow *vs* renal perfusion pressure during venovenous bypass in human orthotopic liver transplantation – *Tranplant Proc* 1993; 25: 2590.

8. Scherer,RU; Giebler,RM; Schmutzler,MJ; Günnicker,FM; Kox,WJ. – Shunt flow and caval pressure gradient during veno-venous bypass in human orthotopic liver transplantation – *Br J Anaesth* 1993; 70: 689-90.

9. Durand,F; Aschehoug,J; Sauvanet,A; Bernuau,J; Benhamou,JP; Erlinger,S; Belghiti,J. – Preservation of renal perfusion and postoperative renal function by side-to-side cavo-caval anastomosis in liver transplant recipients - *Transpl Int* 1995; 8: 407-10.

PROTOCOLO DE PESQUISA:

HEMODINÂMICA SISTÊMICA, PRESSÃO DE PERFUSÃO RENAL E FUNÇÃO RENAL NO TRANSPLANTE DE FÍGADO REALIZADO PELOS MÉTODOS CONVENCIONAL OU *PIGGYBACK*

Identificação: ____

Nome:____

Idade: ______ **RGHC**: ______________________ **Caso n º**: __________

Data do Tx: ____/____/____

Indicação do Tx: ____

Tipo de Cirurgia : ( ) Convencional

( ) *Piggyback*

Critério de Inclusão:

Consentimento pós-informação assinado Sim ( ) Não ( )

Idade maior ou igual a 18 anos Sim ( ) Não ( )

Idade inferior ou igual a 60 anos Sim ( ) Não ( )

Critérios de Exclusão:

Polineuropatia amiloidótica familiar Sim ( ) Não ( )

Retransplante de fígado Sim ( ) Não ( )

Infecção ativa Sim ( ) Não ( )

Impossibilidade de aplicação do método previsto Sim ( ) Não ( )

Anastomose porto-cava Sim ( ) Não ( )

Fígado reduzido Sim ( ) Não ( )

Obs: Serão estudados os pacientes com resposta “sim” a todos os critérios de inclusão e “não” a todos os critérios de exclusão.

Nome: **Caso nº:**

Resultado:

	FASE 1	FASE 2	FASE 3
**DÉBITO CARDÍACO** (L / min)			
**RESISTÊNCIA VASCULAR PERIFÉRICA (NÃO INDEXADA)**			
**PRESSÃO VENOSA CENTRAL** (mmHg)			
**PRESSÃO ARTERIAL MÉDIA** (mmHg)			
**PRESSÃO DE VEIA CAVA INFERIOR** (mmHg)			

fase 1: imediatamente antes da hepatectomia; fase 2: durante a fase anepática; fase 3: uma hora após a reperfusão.

DÉBITO URINÁRIO NA FASE ANEPÁTICA: _____________ ml/kg/minuto

Volume urinário: _____________ ml

Peso do paciente: _____________ Kg

Tempo de coleta: _____________ minutos

FUNÇÃO RENAL:

	pré-op.	1º. PO	5º. PO	30º. PO
**URÉIA SÉRICA**				
**CREATININA SÉRICA**				
**CREATININA URINÁRIA**				
**VOLUME URINÁRIO DE 24 HORAS**				
**DEPURAÇÃO DE CREATININA**				
