# Supplementary material for: Prospective Randomized Trial Comparing Hepatic Venous Outflow and Renal Function after Conventional versus Piggyback Liver Transplantation
Source: PLoS One. 2015 Jun 26;10(6):e0129923. doi: 10.1371/journal.pone.0129923 (PMC4482688; doi:10.1371/journal.pone.0129923)
Supplement: S4 Protocol — (PDF) [file pone.0129923.s005.pdf]

RESEARCH PROTOCOL

Systemic hemodynamics, renal perfusion pressure and renal function after conventional or piggyback liver transplantation.

Responsible investigator: Dr. Paulo Celso Bosco Massarollo

1- Introduction and justification

In the conventional technique of liver transplantation (LTx), the liver is removed *en bloc* with the retrohepatic portion of the inferior vena cava (IVC), which remains clamped below of the diaphragm and above the renal veins during all the anhepatic phase. Interrupting the venous return of the infradiaphragmatic bed determines fall in the arterial blood pressure and venous congestion of the kidneys, reducing the renal perfusion pressure (RPP).[1] In order to overcome the consequences of this maneuver, a temporary venovenous bypass (VVB) driven by a centrifugal pump is usually used, allowing diversion of the IVC blood flow to the superior vena cava.[2] Despite these advantages, VVB presents risks such as air and thrombotic pulmonary embolism,[1,3] and lymphatic fistulas in the axillary and inguinal regions,[4] where incisions for the placement of catheters are performed. In addition, the cost of the procedure is raised, due to necessity of specific catheters, of a special centrifugal pump machine and a dedicated equipment operator. More recently, the piggyback method of LTx was described, in which the diseased liver is removed with preservation of the retrohepatic portion of the IVC.[5] Thus, flow through IVC can be maintained, eliminating the need for VVB and its inconveniences.[6]

Although the two surgical methods intend to prevent hemodynamic derangements secondary to IVC clamping, this goal is achieved just partially in both. During VVB there is an inverse correlation between the extra-corporeal circuit blood flow and RPP.[(7)] It means that this procedure is less efficient when low circuit flow occurs.[(8)] Similarly, in the piggyback method, clamping of the hepatic veins can involve side clamping of recipient IVC, and partially impair the venous return. In fact, an increased IVC pressure (IVCP) is described during the anhepatic phase of piggyback LTx, although RPP is preserved due to a concomitant rise in mean arterial pressure (MAP).[(9)] To the best of our knowledge, no previous study compared by means of a clinical randomized prospective trial the piggyback or conventional with VVB LTx regarding the efficiency of IVC decompression and the hemodynamic and renal consequences of partial venous return impairment.

2- Aim

To compare systemic hemodynamics, RPP and postoperative renal function in patients submitted to conventional with VVB or piggyback LTx.

3- Patients and methods

Forty-two LTx candidates, who will participate of the research protocols ***"Evaluation of inflammatory cytokines in two operative methods of liver transplantation: conventional or piggyback"*** and ***"Evaluation of bacterial***

translocation in liver transplantation”, and that agree to also participating of this research, will be prospectively studied. These protocols, which have been already analyzed and approved by the Department of Surgery of FMUSP (Faculdade de Medicina da Universidade de São Paulo) and by CAPPesq (Ethics Committee of the Institution - Comissão de Ética para Análise de Projetos de Pesquisa do Hospital das Clínicas da Faculdade de Medicina da Universidade de São Paulo), admitted randomization of both genders patients, with age between 18 and 60 years, to two groups: LTx performed using the conventional or the piggyback method. Randomization will be conducted immediately before the surgery, by using a table of random numbers. Exclusion criteria include “familial amyloidotic polyneuropathy” diagnosis, liver retransplantation, active infection, technical impossibility of accomplishment of the surgical method predicted by randomization, necessity of temporary porto-caval anatomosis, and reduced (partial) liver graft. As the patients will be shared with these two studies, the same criteria will be adopted in the current protocol.

According to the routine of our service, all LTx recipients are submitted to hemodynamic monitoring using a Swan-Ganz catheter. This catheter will be used for measurement of the cardiac output and the central venous pressure. MAP is continuously registered in all cases (routine procedure). The values of the cardiac output, the central venous pressure and MAP will be used for calculation of the systemic vascular resistance index. Intraoperative hemodynamic evaluations will be performed at 3 moments: immediately before total hepatectomy (phase 1); during the anhepatic phase (phase 2); one hour after the graft reperfusion (phase 3). Percutaneous access to the left femoral vein will be obtained using the Seldinger technique. A 7 French

angiographic catheter with a lateral orifice in the distal end will be introduced and located into the IVC, at to level of the renal veins entrance. The surgeon will confirm the positioning of the catheter by palpation. This catheter will be used for measurement of the IVCP. RPP will be evaluated by the difference between MAP and the IVCP. This measurement will be performed at 3 moments, simultaneously with systemic hemodynamics evaluation.

Pre- and postoperative renal function will be evaluated by using serum dosages of urea and creatinine and by calculation of 24-hour creatinine clearance. The preoperative evaluation will be performed at least 2 months before LTx date. This evaluation will be repeated in 1st, 5th and 30th postoperative days. During the anhepatic phase, diuresis will be registered in mL/kg/minute.

Numerical results will be submitted to the test of Bartlett to evaluate the homogeneity of the variances. If the variances are uniforms, the groups will be compared by means of analysis of variance (ANOVA). When this does not occur, the test of Kruskal-Wallis will be used.

The sample size would be sufficient to detect a difference equal to or higher than 3.08 mmHg between the two surgical groups regarding anhepatic phase RPP mean values, fixing type I and II errors in 5% (alpha error = beta error = 0.05) and considering an expected standard deviation of 2.7 mmHg.[8]

References

1. Griffith,BP; Shaw,BWJr; Hardesty,RL;Iwatsuki,S; Bahnson,HT;Starzl,TE. – Veno-venous bypass without systemic anticoagulation for transplantation of human liver. *Surg Gynecol Obstet* 1985, 160: 271-72.

2. Shaw, B; Martin, D; Marquez,J. – Venous bypass clinical liver transplantation. *Ann Surg.* 1984; 200:524-9.

3. Fleitas, MG; Casanova, D; Martino, E; Maestre, JM; Herrera, L; Hernanz, F; Rabanal, JM; Pulgar, S; Solares, G. – Could the piggyback operation in liver transplantation be routinely used ? *Arch Surg.* 1994;129:842-5.

4. Stieber, AC. – One surgeon's experience with the piggyback versus the standard technique in orthotopic liver transplantation: is one better than the other? *Hepato-Gastroenterology.*1995; 42:403-5.

5. Tzakis, A; Todo, S; Starzl, T. – Orthotopic liver transplantation with preservation of the inferior vena cava. *Ann Surg.*1989; 210:649-55.

6. Belghiti, J; Panis, Y; Sauvanet, A; Gayet, B; Fekete, F. – A new technique of caval anastomosis during orthotopic liver transplantation without inferior vena cava occlusion. *Surg Gynecol Obstet* 1992; 175:270-72.

7. Scherer,RU; Giebler,RM; Schmutzler,MJ;Erhard,J; Lange,R; Kox,WJ. – Shuntflow *vs* renal perfusion pressure during venovenous bypass in human orthotopic liver transplantation. *Tranplant Proc* 1993; 25: 2590.

8. Scherer,RU; Giebler,RM; Schmutzler,MJ; Günnicker,FM; Kox,WJ. – Shunt flow and caval pressure gradient during veno-venous bypass in human orthotopic liver transplantation. *Br J Anaesth* 1993; 70: 689-90.

9. Durand,F; Aschehoug,J; Sauvanet,A; Bernuau,J; Benhamou,JP; Erlinger,S; Belghiti,J. – Preservation of renal perfusion and postoperative renal function by side-to-side cavo-caval anastomosis in liver transplant recipients. *Transpl Int* 1995; 8: 407-10.
